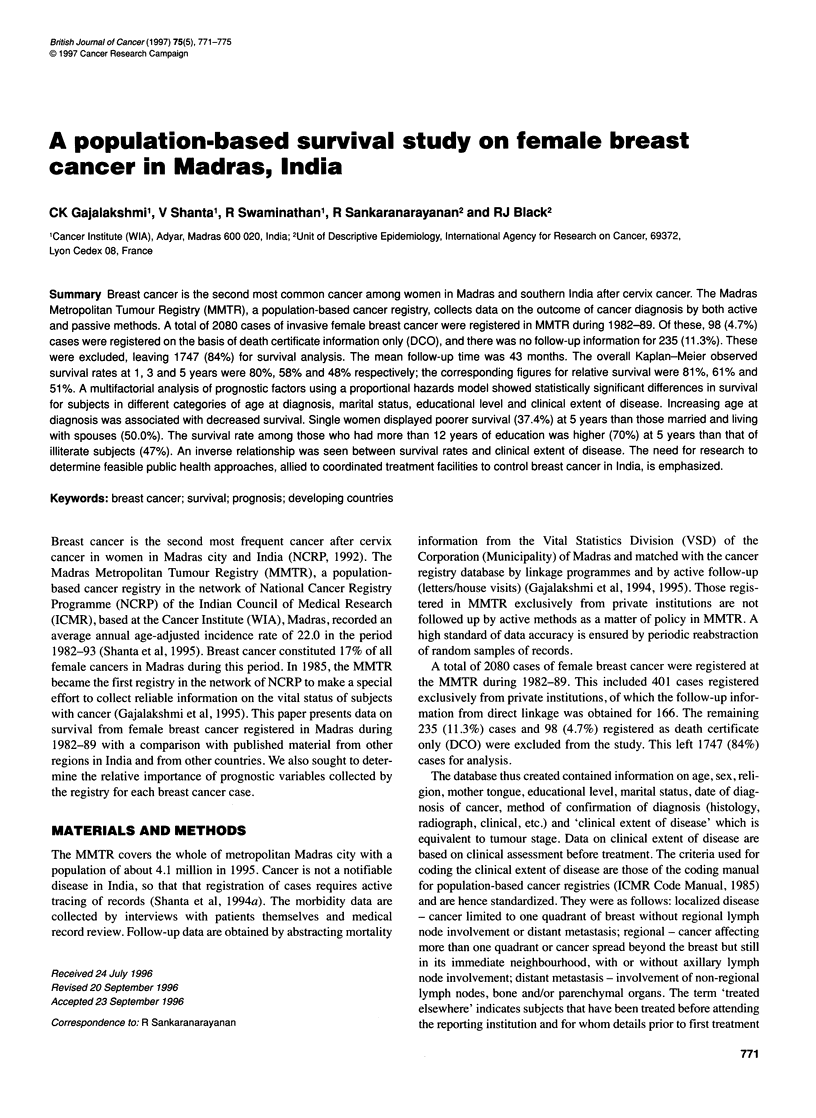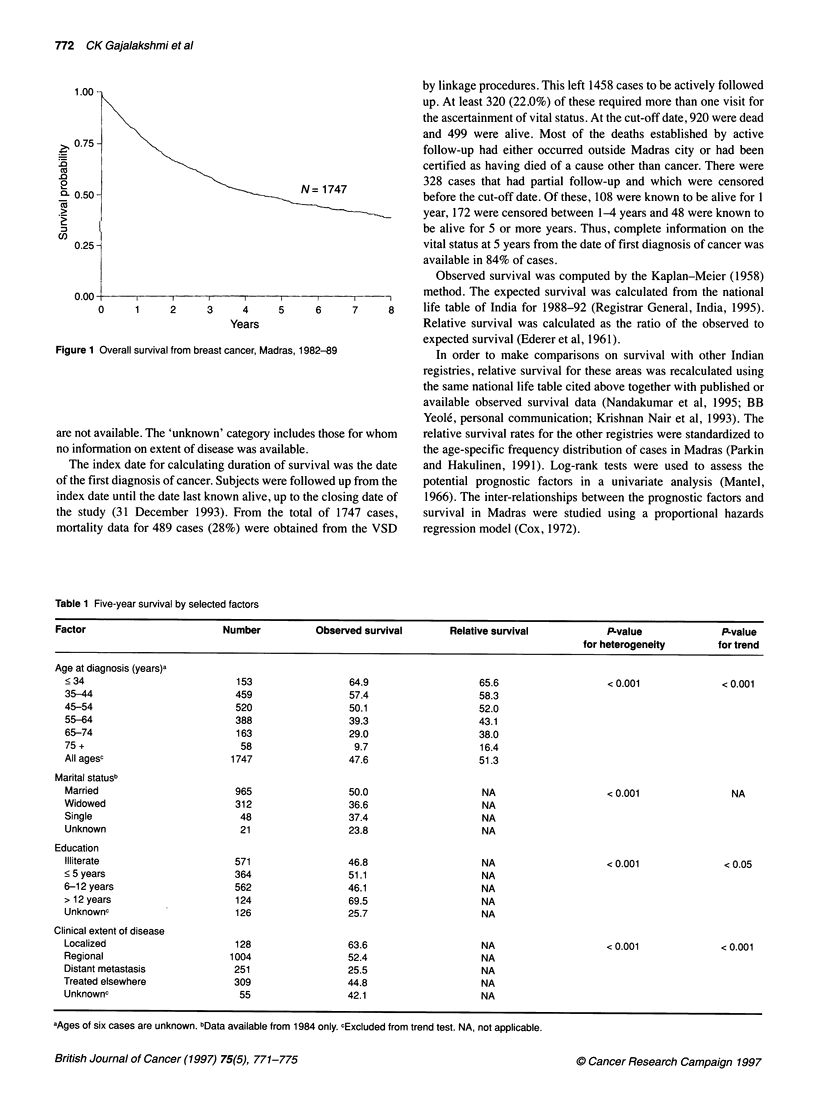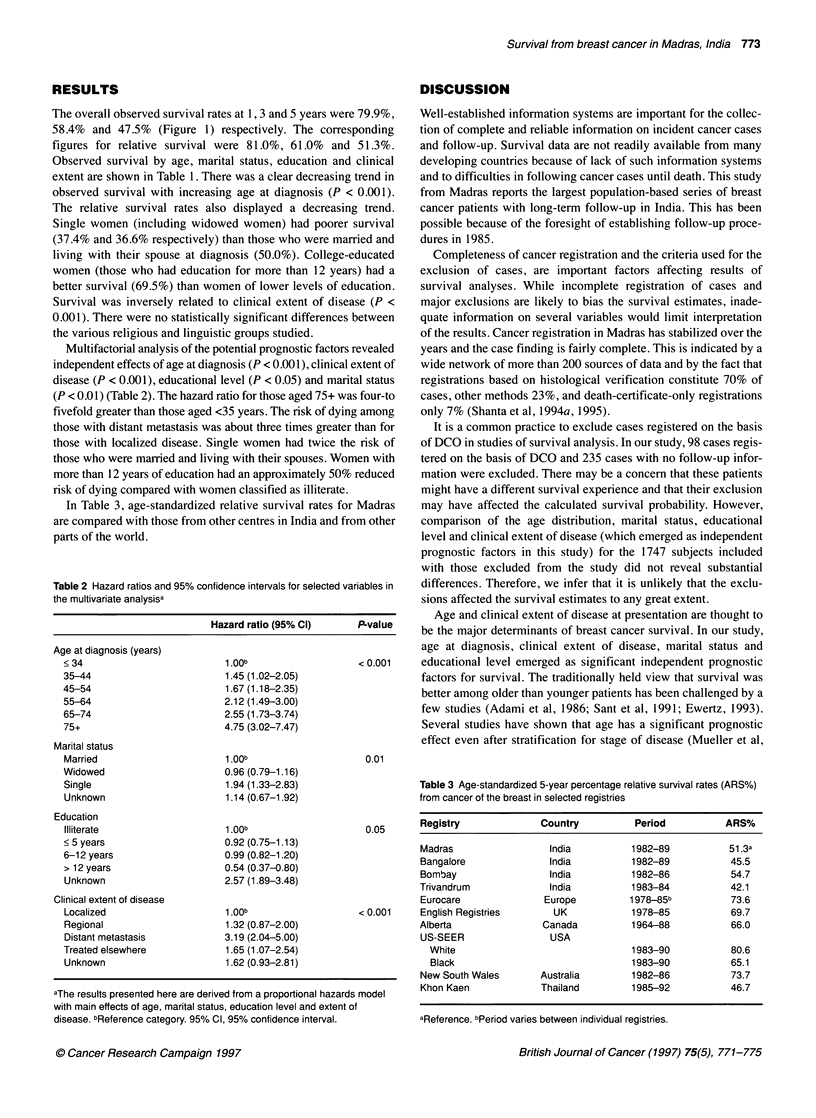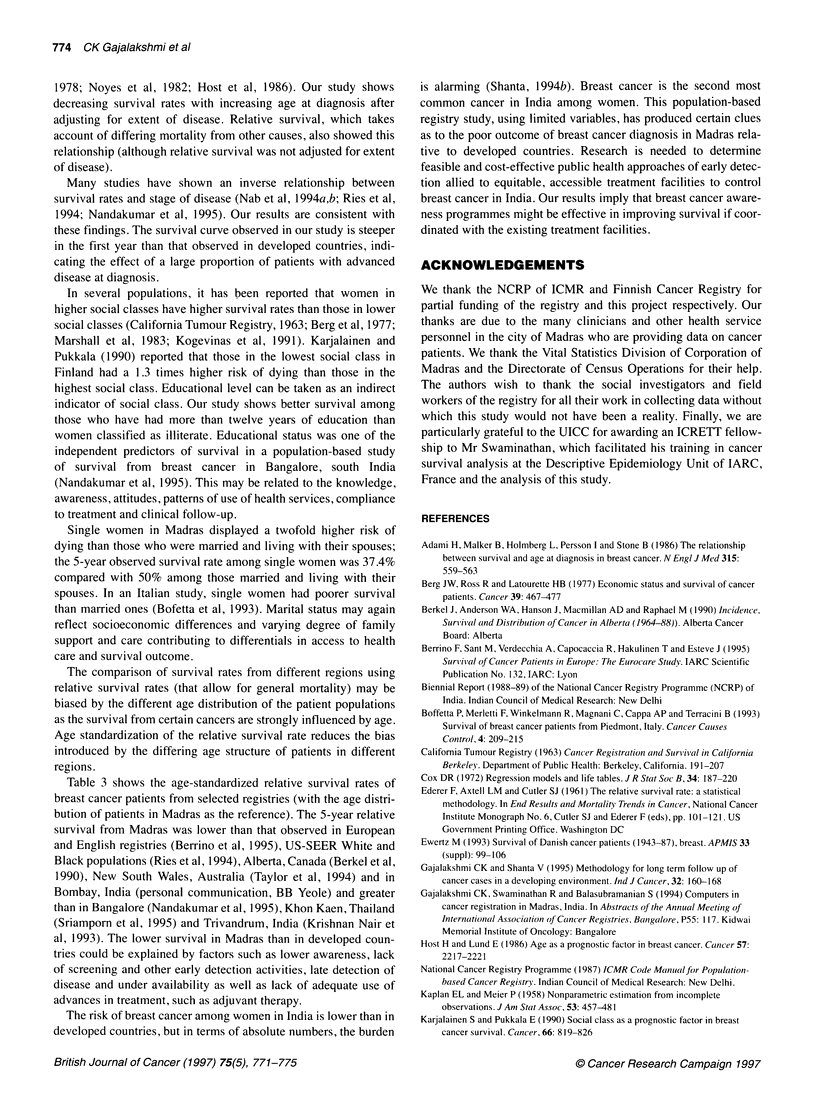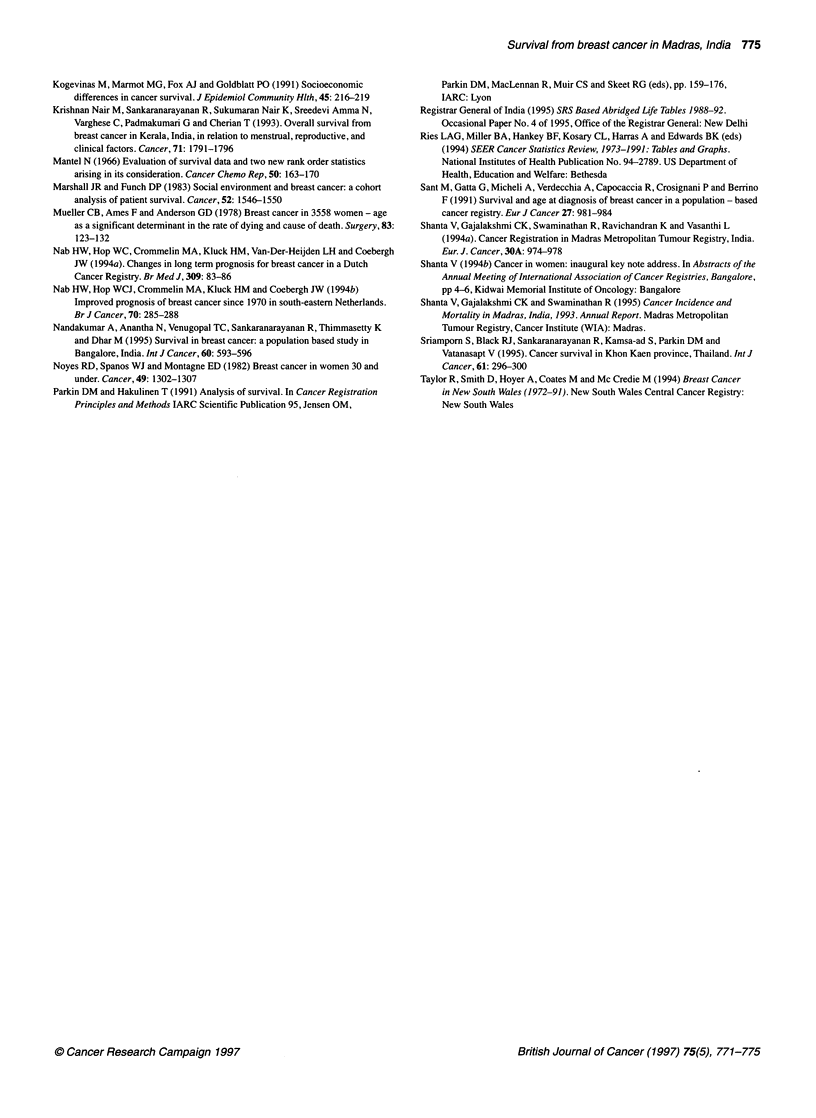# A population-based survival study on female breast cancer in Madras, India.

**DOI:** 10.1038/bjc.1997.137

**Published:** 1997

**Authors:** C. K. Gajalakshmi, V. Shanta, R. Swaminathan, R. Sankaranarayanan, R. J. Black

**Affiliations:** Cancer Institute (WIA), Adyar, Madras, India.

## Abstract

Breast cancer is the second most common cancer among women in Madras and southern India after cervix cancer. The Madras Metropolitan Tumour Registry (MMTR), a population-based cancer registry, collects data on the outcome of cancer diagnosis by both active and passive methods. A total of 2080 cases of invasive female breast cancer were registered in MMTR during 1982-89. Of these, 98 (4.7%) cases were registered on the basis of death certificate information only (DCO), and there was no follow-up information for 235 (11.3%). These were excluded, leaving 1747 (84%) for survival analysis. The mean follow-up time was 43 months. The overall Kaplan-Meier observed survival rates at 1, 3 and 5 years were 80%, 58% and 48% respectively; the corresponding figures for relative survival were 81%, 61% and 51%. A multifactorial analysis of prognostic factors using a proportional hazards model showed statistically significant differences in survival for subjects in different categories of age at diagnosis, marital status, educational level and clinical extent of disease. Increasing age at diagnosis was associated with decreased survival. Single women displayed poorer survival (37.4%) at 5 years than those married and living with spouses (50.0%). The survival rate among those who had more than 12 years of education was higher (70%) at 5 years than that of illiterate subjects (47%). An inverse relationship was seen between survival rates and clinical extent of disease. The need for research to determine feasible public health approaches, allied to coordinated treatment facilities to control breast cancer in India, is emphasized.


British Journal of Cancer (1997) 75(5), 771–775
© 1997 Cancer Research Campaign

### A population-based survival study on female breast cancer in Madras, India

**CK Gajalakshmi[1], V Shanta[1], R Swaminathan[1], R Sankaranarayanan[2] and RJ Black[2]**

[1]Cancer Institute (WIA), Adyar, Madras 600 020, India; [2]Unit of Descriptive Epidemiology, International Agency for Research on Cancer, 69372, Lyon Cedex 08, France

**Summary** Breast cancer is the second most common cancer among women in Madras and southern India after cervix cancer. The Madras Metropolitan Tumour Registry (MMTR), a population-based cancer registry, collects data on the outcome of cancer diagnosis by both active and passive methods. A total of 2080 cases of invasive female breast cancer were registered in MMTR during 1982–89. Of these, 98 (4.7%) cases were registered on the basis of death certificate information only (DCO), and there was no follow-up information for 235 (11.3%). These were excluded, leaving 1747 (84%) for survival analysis. The mean follow-up time was 43 months. The overall Kaplan–Meier observed survival rates at 1, 3 and 5 years were 80%, 58% and 48% respectively; the corresponding figures for relative survival were 81%, 61% and 51%. A multifactorial analysis of prognostic factors using a proportional hazards model showed statistically significant differences in survival for subjects in different categories of age at diagnosis, marital status, educational level and clinical extent of disease. Increasing age at diagnosis was associated with decreased survival. Single women displayed poorer survival (37.4%) at 5 years than those married and living with spouses (50.0%). The survival rate among those who had more than 12 years of education was higher (70%) at 5 years than that of illiterate subjects (47%). An inverse relationship was seen between survival rates and clinical extent of disease. The need for research to determine feasible public health approaches, allied to coordinated treatment facilities to control breast cancer in India, is emphasized.

**Keywords:** breast cancer; survival; prognosis; developing countries

Breast cancer is the second most frequent cancer after cervix cancer in women in Madras city and India (NCRP, 1992). The Madras Metropolitan Tumour Registry (MMTR), a population-based cancer registry in the network of National Cancer Registry Programme (NCRP) of the Indian Council of Medical Research (ICMR), based at the Cancer Institute (WIA), Madras, recorded an average annual age-adjusted incidence rate of 22.0 in the period 1982–93 (Shanta et al, 1995). Breast cancer constituted 17% of all female cancers in Madras during this period. In 1985, the MMTR became the first registry in the network of NCRP to make a special effort to collect reliable information on the vital status of subjects with cancer (Gajalakshmi et al, 1995). This paper presents data on survival from female breast cancer registered in Madras during 1982–89 with a comparison with published material from other regions in India and from other countries. We also sought to determine the relative importance of prognostic variables collected by the registry for each breast cancer case.

### MATERIALS AND METHODS

The MMTR covers the whole of metropolitan Madras city with a population of about 4.1 million in 1995. Cancer is not a notifiable disease in India, so that that registration of cases requires active tracing of records (Shanta et al, 1994*a*). The morbidity data are collected by interviews with patients themselves and medical record review. Follow-up data are obtained by abstracting mortality information from the Vital Statistics Division (VSD) of the Corporation (Municipality) of Madras and matched with the cancer registry database by linkage programmes and by active follow-up (letters/house visits) (Gajalakshmi et al, 1994, 1995). Those registered in MMTR exclusively from private institutions are not followed up by active methods as a matter of policy in MMTR. A high standard of data accuracy is ensured by periodic reabstraction of random samples of records.

A total of 2080 cases of female breast cancer were registered at the MMTR during 1982–89. This included 401 cases registered exclusively from private institutions, of which the follow-up information from direct linkage was obtained for 166. The remaining 235 (11.3%) cases and 98 (4.7%) registered as death certificate only (DCO) were excluded from the study. This left 1747 (84%) cases for analysis.

The database thus created contained information on age, sex, religion, mother tongue, educational level, marital status, date of diagnosis of cancer, method of confirmation of diagnosis (histology, radiograph, clinical, etc.) and 'clinical extent of disease' which is equivalent to tumour stage. Data on clinical extent of disease are based on clinical assessment before treatment. The criteria used for coding the clinical extent of disease are those of the coding manual for population-based cancer registries (ICMR Code Manual, 1985) and are hence standardized. They were as follows: localized disease – cancer limited to one quadrant of breast without regional lymph node involvement or distant metastasis; regional – cancer affecting more than one quadrant or cancer spread beyond the breast but still in its immediate neighbourhood, with or without axillary lymph node involvement; distant metastasis – involvement of non-regional lymph nodes, bone and/or parenchymal organs. The term 'treated elsewhere' indicates subjects that have been treated before attending the reporting institution and for whom details prior to first treatment

*Received 24 July 1996*
*Revised 20 September 1996*
*Accepted 23 September 1996*

*Correspondence to:* R Sankaranarayanan

**Figure 1** Overall survival from breast cancer, Madras, 1982–89

are not available. The 'unknown' category includes those for whom no information on extent of disease was available.

The index date for calculating duration of survival was the date of the first diagnosis of cancer. Subjects were followed up from the index date until the date last known alive, up to the closing date of the study (31 December 1993). From the total of 1747 cases, mortality data for 489 cases (28%) were obtained from the VSD by linkage procedures. This left 1458 cases to be actively followed up. At least 320 (22.0%) of these required more than one visit for the ascertainment of vital status. At the cut-off date, 920 were dead and 499 were alive. Most of the deaths established by active follow-up had either occurred outside Madras city or had been certified as having died of a cause other than cancer. There were 328 cases that had partial follow-up and which were censored before the cut-off date. Of these, 108 were known to be alive for 1 year, 172 were censored between 1–4 years and 48 were known to be alive for 5 or more years. Thus, complete information on the vital status at 5 years from the date of first diagnosis of cancer was available in 84% of cases.

Observed survival was computed by the Kaplan–Meier (1958) method. The expected survival was calculated from the national life table of India for 1988–92 (Registrar General, India, 1995). Relative survival was calculated as the ratio of the observed to expected survival (Ederer et al, 1961).

In order to make comparisons on survival with other Indian registries, relative survival for these areas was recalculated using the same national life table cited above together with published or available observed survival data (Nandakumar et al, 1995; BB Yeolé, personal communication; Krishnan Nair et al, 1993). The relative survival rates for the other registries were standardized to the age-specific frequency distribution of cases in Madras (Parkin and Hakulinen, 1991). Log-rank tests were used to assess the potential prognostic factors in a univariate analysis (Mantel, 1966). The inter-relationships between the prognostic factors and survival in Madras were studied using a proportional hazards regression model (Cox, 1972).

**Table 1** Five-year survival by selected factors

| Factor | Number | Observed survival | Relative survival | *P*-value for heterogeneity | *P*-value for trend |
|---|---|---|---|---|---|
| Age at diagnosis (years)[a] | | | | | |
| ≤ 34 | 153 | 64.9 | 65.6 | < 0.001 | < 0.001 |
| 35–44 | 459 | 57.4 | 58.3 | | |
| 45–54 | 520 | 50.1 | 52.0 | | |
| 55–64 | 388 | 39.3 | 43.1 | | |
| 65–74 | 163 | 29.0 | 38.0 | | |
| 75 + | 58 | 9.7 | 16.4 | | |
| All ages[c] | 1747 | 47.6 | 51.3 | | |
| Marital status[b] | | | | | |
| Married | 965 | 50.0 | NA | < 0.001 | NA |
| Widowed | 312 | 36.6 | NA | | |
| Single | 48 | 37.4 | NA | | |
| Unknown | 21 | 23.8 | NA | | |
| Education | | | | | |
| Illiterate | 571 | 46.8 | NA | < 0.001 | < 0.05 |
| ≤ 5 years | 364 | 51.1 | NA | | |
| 6–12 years | 562 | 46.1 | NA | | |
| > 12 years | 124 | 69.5 | NA | | |
| Unknown[c] | 126 | 25.7 | NA | | |
| Clinical extent of disease | | | | | |
| Localized | 128 | 63.6 | NA | < 0.001 | < 0.001 |
| Regional | 1004 | 52.4 | NA | | |
| Distant metastasis | 251 | 25.5 | NA | | |
| Treated elsewhere | 309 | 44.8 | NA | | |
| Unknown[c] | 55 | 42.1 | NA | | |

[a]Ages of six cases are unknown. [b]Data available from 1984 only. [c]Excluded from trend test. NA, not applicable.

© Cancer Research Campaign 1997

### RESULTS

The overall observed survival rates at 1, 3 and 5 years were 79.9%, 58.4% and 47.5% (Figure 1) respectively. The corresponding figures for relative survival were 81.0%, 61.0% and 51.3%. Observed survival by age, marital status, education and clinical extent are shown in Table 1. There was a clear decreasing trend in observed survival with increasing age at diagnosis ($P < 0.001$). The relative survival rates also displayed a decreasing trend. Single women (including widowed women) had poorer survival (37.4% and 36.6% respectively) than those who were married and living with their spouse at diagnosis (50.0%). College-educated women (those who had education for more than 12 years) had a better survival (69.5%) than women of lower levels of education. Survival was inversely related to clinical extent of disease ($P < 0.001$). There were no statistically significant differences between the various religious and linguistic groups studied.

Multifactorial analysis of the potential prognostic factors revealed independent effects of age at diagnosis ($P < 0.001$), clinical extent of disease ($P < 0.001$), educational level ($P < 0.05$) and marital status ($P < 0.01$) (Table 2). The hazard ratio for those aged 75+ was four-to fivefold greater than those aged <35 years. The risk of dying among those with distant metastasis was about three times greater than for those with localized disease. Single women had twice the risk of those who were married and living with their spouses. Women with more than 12 years of education had an approximately 50% reduced risk of dying compared with women classified as illiterate.

In Table 3, age-standardized relative survival rates for Madras are compared with those from other centres in India and from other parts of the world.

**Table 2** Hazard ratios and 95% confidence intervals for selected variables in the multivariate analysis[a]

| | Hazard ratio (95% CI) | P-value |
|---|---|---|
| Age at diagnosis (years) | | |
| ≤ 34 | 1.00[b] | < 0.001 |
| 35–44 | 1.45 (1.02–2.05) | |
| 45–54 | 1.67 (1.18–2.35) | |
| 55–64 | 2.12 (1.49–3.00) | |
| 65–74 | 2.55 (1.73–3.74) | |
| 75+ | 4.75 (3.02–7.47) | |
| Marital status | | |
| Married | 1.00[b] | 0.01 |
| Widowed | 0.96 (0.79–1.16) | |
| Single | 1.94 (1.33–2.83) | |
| Unknown | 1.14 (0.67–1.92) | |
| Education | | |
| Illiterate | 1.00[b] | 0.05 |
| ≤ 5 years | 0.92 (0.75–1.13) | |
| 6–12 years | 0.99 (0.82–1.20) | |
| > 12 years | 0.54 (0.37–0.80) | |
| Unknown | 2.57 (1.89–3.48) | |
| Clinical extent of disease | | |
| Localized | 1.00[b] | < 0.001 |
| Regional | 1.32 (0.87–2.00) | |
| Distant metastasis | 3.19 (2.04–5.00) | |
| Treated elsewhere | 1.65 (1.07–2.54) | |
| Unknown | 1.62 (0.93–2.81) | |

[a]The results presented here are derived from a proportional hazards model with main effects of age, marital status, education level and extent of disease. [b]Reference category. 95% CI, 95% confidence interval.

### DISCUSSION

Well-established information systems are important for the collection of complete and reliable information on incident cancer cases and follow-up. Survival data are not readily available from many developing countries because of lack of such information systems and to difficulties in following cancer cases until death. This study from Madras reports the largest population-based series of breast cancer patients with long-term follow-up in India. This has been possible because of the foresight of establishing follow-up procedures in 1985.

Completeness of cancer registration and the criteria used for the exclusion of cases, are important factors affecting results of survival analyses. While incomplete registration of cases and major exclusions are likely to bias the survival estimates, inadequate information on several variables would limit interpretation of the results. Cancer registration in Madras has stabilized over the years and the case finding is fairly complete. This is indicated by a wide network of more than 200 sources of data and by the fact that registrations based on histological verification constitute 70% of cases, other methods 23%, and death-certificate-only registrations only 7% (Shanta et al, 1994*a*, 1995).

It is a common practice to exclude cases registered on the basis of DCO in studies of survival analysis. In our study, 98 cases registered on the basis of DCO and 235 cases with no follow-up information were excluded. There may be a concern that these patients might have a different survival experience and that their exclusion may have affected the calculated survival probability. However, comparison of the age distribution, marital status, educational level and clinical extent of disease (which emerged as independent prognostic factors in this study) for the 1747 subjects included with those excluded from the study did not reveal substantial differences. Therefore, we infer that it is unlikely that the exclusions affected the survival estimates to any great extent.

Age and clinical extent of disease at presentation are thought to be the major determinants of breast cancer survival. In our study, age at diagnosis, clinical extent of disease, marital status and educational level emerged as significant independent prognostic factors for survival. The traditionally held view that survival was better among older than younger patients has been challenged by a few studies (Adami et al, 1986; Sant et al, 1991; Ewertz, 1993). Several studies have shown that age has a significant prognostic effect even after stratification for stage of disease (Mueller et al,

**Table 3** Age-standardized 5-year percentage relative survival rates (ARS%) from cancer of the breast in selected registries

| Registry | Country | Period | ARS% |
|---|---|---|---|
| Madras | India | 1982–89 | 51.3[a] |
| Bangalore | India | 1982–89 | 45.5 |
| Bombay | India | 1982–86 | 54.7 |
| Trivandrum | India | 1983–84 | 42.1 |
| Eurocare | Europe | 1978–85[b] | 73.6 |
| English Registries | UK | 1978–85 | 69.7 |
| Alberta | Canada | 1964–88 | 66.0 |
| US-SEER | USA | | |
| White | | 1983–90 | 80.6 |
| Black | | 1983–90 | 65.1 |
| New South Wales | Australia | 1982–86 | 73.7 |
| Khon Kaen | Thailand | 1985–92 | 46.7 |

[a]Reference. [b]Period varies between individual registries.

© Cancer Research Campaign 1997

1978; Noyes et al, 1982; Host et al, 1986). Our study shows decreasing survival rates with increasing age at diagnosis after adjusting for extent of disease. Relative survival, which takes account of differing mortality from other causes, also showed this relationship (although relative survival was not adjusted for extent of disease).

Many studies have shown an inverse relationship between survival rates and stage of disease (Nab et al, 1994*a*,*b*; Ries et al, 1994; Nandakumar et al, 1995). Our results are consistent with these findings. The survival curve observed in our study is steeper in the first year than that observed in developed countries, indicating the effect of a large proportion of patients with advanced disease at diagnosis.

In several populations, it has been reported that women in higher social classes have higher survival rates than those in lower social classes (California Tumour Registry, 1963; Berg et al, 1977; Marshall et al, 1983; Kogevinas et al, 1991). Karjalainen and Pukkala (1990) reported that those in the lowest social class in Finland had a 1.3 times higher risk of dying than those in the highest social class. Educational level can be taken as an indirect indicator of social class. Our study shows better survival among those who have had more than twelve years of education than women classified as illiterate. Educational status was one of the independent predictors of survival in a population-based study of survival from breast cancer in Bangalore, south India (Nandakumar et al, 1995). This may be related to the knowledge, awareness, attitudes, patterns of use of health services, compliance to treatment and clinical follow-up.

Single women in Madras displayed a twofold higher risk of dying than those who were married and living with their spouses; the 5-year observed survival rate among single women was 37.4% compared with 50% among those married and living with their spouses. In an Italian study, single women had poorer survival than married ones (Bofetta et al, 1993). Marital status may again reflect socioeconomic differences and varying degree of family support and care contributing to differentials in access to health care and survival outcome.

The comparison of survival rates from different regions using relative survival rates (that allow for general mortality) may be biased by the different age distribution of the patient populations as the survival from certain cancers are strongly influenced by age. Age standardization of the relative survival rate reduces the bias introduced by the differing age structure of patients in different regions.

Table 3 shows the age-standardized relative survival rates of breast cancer patients from selected registries (with the age distribution of patients in Madras as the reference). The 5-year relative survival from Madras was lower than that observed in European and English registries (Berrino et al, 1995), US-SEER White and Black populations (Ries et al, 1994), Alberta, Canada (Berkel et al, 1990), New South Wales, Australia (Taylor et al, 1994) and in Bombay, India (personal communication, BB Yeole) and greater than in Bangalore (Nandakumar et al, 1995), Khon Kaen, Thailand (Sriamporn et al, 1995) and Trivandrum, India (Krishnan Nair et al, 1993). The lower survival in Madras than in developed countries could be explained by factors such as lower awareness, lack of screening and other early detection activities, late detection of disease and under availability as well as lack of adequate use of advances in treatment, such as adjuvant therapy.

The risk of breast cancer among women in India is lower than in developed countries, but in terms of absolute numbers, the burden is alarming (Shanta, 1994*b*). Breast cancer is the second most common cancer in India among women. This population-based registry study, using limited variables, has produced certain clues as to the poor outcome of breast cancer diagnosis in Madras relative to developed countries. Research is needed to determine feasible and cost-effective public health approaches of early detection allied to equitable, accessible treatment facilities to control breast cancer in India. Our results imply that breast cancer awareness programmes might be effective in improving survival if coordinated with the existing treatment facilities.

### ACKNOWLEDGEMENTS

We thank the NCRP of ICMR and Finnish Cancer Registry for partial funding of the registry and this project respectively. Our thanks are due to the many clinicians and other health service personnel in the city of Madras who are providing data on cancer patients. We thank the Vital Statistics Division of Corporation of Madras and the Directorate of Census Operations for their help. The authors wish to thank the social investigators and field workers of the registry for all their work in collecting data without which this study would not have been a reality. Finally, we are particularly grateful to the UICC for awarding an ICRETT fellowship to Mr Swaminathan, which facilitated his training in cancer survival analysis at the Descriptive Epidemiology Unit of IARC, France and the analysis of this study.

### REFERENCES

Adami H, Malker B, Holmberg L, Persson I and Stone B (1986) The relationship between survival and age at diagnosis in breast cancer. *N Engl J Med* **315**: 559–563

Berg JW, Ross R and Latourette HB (1977) Economic status and survival of cancer patients. *Cancer* **39**: 467–477

Berkel J, Anderson WA, Hanson J, Macmillan AD and Raphael M (1990) *Incidence, Survival and Distribution of Cancer in Alberta (1964–88)*. Alberta Cancer Board: Alberta

Berrino F, Sant M, Verdecchia A, Capocaccia R, Hakulinen T and Esteve J (1995) *Survival of Cancer Patients in Europe: The Eurocare Study*. IARC Scientific Publication No. 132, IARC: Lyon

Biennial Report (1988–89) of the National Cancer Registry Programme (NCRP) of India. Indian Council of Medical Research: New Delhi

Boffetta P, Merletti F, Winkelmann R, Magnani C, Cappa AP and Terracini B (1993) Survival of breast cancer patients from Piedmont, Italy. *Cancer Causes Control*, **4**: 209–215

California Tumour Registry (1963) *Cancer Registration and Survival in California Berkeley*. Department of Public Health: Berkeley, California. 191–207

Cox DR (1972) Regression models and life tables. *J R Stat Soc B*, **34**: 187–220

Ederer F, Axtell LM and Cutler SJ (1961) The relative survival rate: a statistical methodology. In *End Results and Mortality Trends in Cancer*, National Cancer Institute Monograph No. 6, Cutler SJ and Ederer F (eds), pp. 101–121. US Government Printing Office. Washington DC

Ewertz M (1993) Survival of Danish cancer patients (1943–87), breast. *APMIS* **33** (suppl): 99–106

Gajalakshmi CK and Shanta V (1995) Methodology for long term follow up of cancer cases in a developing environment. *Ind J Cancer*, **32**: 160–168

Gajalakshmi CK, Swaminathan R and Balasubramanian S (1994) Computers in cancer registration in Madras, India. In *Abstracts of the Annual Meeting of International Association of Cancer Registries, Bangalore*, P55: 117. Kidwai Memorial Institute of Oncology: Bangalore

Host H and Lund E (1986) Age as a prognostic factor in breast cancer. *Cancer* **57**: 2217–2221

National Cancer Registry Programme (1987) *ICMR Code Manual for Population-based Cancer Registry*. Indian Council of Medical Research: New Delhi.

Kaplan EL and Meier P (1958) Nonparametric estimation from incomplete observations. *J Am Stat Assoc*, **53**: 457–481

Karjalainen S and Pukkala E (1990) Social class as a prognostic factor in breast cancer survival. *Cancer*, **66**: 819–826

© Cancer Research Campaign 1997

Kogevinas M, Marmot MG, Fox AJ and Goldblatt PO (1991) Socioeconomic differences in cancer survival. *J Epidemiol Community Hlth*, **45**: 216–219

Krishnan Nair M, Sankaranarayanan R, Sukumaran Nair K, Sreedevi Amma N, Varghese C, Padmakumari G and Cherian T (1993). Overall survival from breast cancer in Kerala, India, in relation to menstrual, reproductive, and clinical factors. *Cancer*, **71**: 1791–1796

Mantel N (1966) Evaluation of survival data and two new rank order statistics arising in its consideration. *Cancer Chemo Rep*, **50**: 163–170

Marshall JR and Funch DP (1983) Social environment and breast cancer: a cohort analysis of patient survival. *Cancer*, **52**: 1546–1550

Mueller CB, Ames F and Anderson GD (1978) Breast cancer in 3558 women – age as a significant determinant in the rate of dying and cause of death. *Surgery*, **83**: 123–132

Nab HW, Hop WC, Crommelin MA, Kluck HM, Van-Der-Heijden LH and Coebergh JW (1994*a*). Changes in long term prognosis for breast cancer in a Dutch Cancer Registry. *Br Med J*, **309**: 83–86

Nab HW, Hop WCJ, Crommelin MA, Kluck HM and Coebergh JW (1994*b*) Improved prognosis of breast cancer since 1970 in south-eastern Netherlands. *Br J Cancer*, **70**: 285–288

Nandakumar A, Anantha N, Venugopal TC, Sankaranarayanan R, Thimmasetty K and Dhar M (1995) Survival in breast cancer: a population based study in Bangalore, India. *Int J Cancer*, **60**: 593–596

Noyes RD, Spanos WJ and Montagne ED (1982) Breast cancer in women 30 and under. *Cancer*, **49**: 1302–1307

Parkin DM and Hakulinen T (1991) Analysis of survival. In *Cancer Registration Principles and Methods* IARC Scientific Publication 95, Jensen OM, Parkin DM, MacLennan R, Muir CS and Skeet RG (eds), pp. 159–176, IARC: Lyon

Registrar General of India (1995) *SRS Based Abridged Life Tables 1988–92*. Occasional Paper No. 4 of 1995, Office of the Registrar General: New Delhi

Ries LAG, Miller BA, Hankey BF, Kosary CL, Harras A and Edwards BK (eds) (1994) *SEER Cancer Statistics Review, 1973–1991: Tables and Graphs*. National Institutes of Health Publication No. 94–2789. US Department of Health, Education and Welfare: Bethesda

Sant M, Gatta G, Micheli A, Verdecchia A, Capocaccia R, Crosignani P and Berrino F (1991) Survival and age at diagnosis of breast cancer in a population – based cancer registry. *Eur J Cancer* **27**: 981–984

Shanta V, Gajalakshmi CK, Swaminathan R, Ravichandran K and Vasanthi L (1994*a*). Cancer Registration in Madras Metropolitan Tumour Registry, India. *Eur. J. Cancer*, **30A**: 974–978

Shanta V (1994*b*) Cancer in women: inaugural key note address. In *Abstracts of the Annual Meeting of International Association of Cancer Registries, Bangalore*, pp 4–6, Kidwai Memorial Institute of Oncology: Bangalore

Shanta V, Gajalakshmi CK and Swaminathan R (1995) *Cancer Incidence and Mortality in Madras, India, 1993. Annual Report*. Madras Metropolitan Tumour Registry, Cancer Institute (WIA): Madras.

Sriamporn S, Black RJ, Sankaranarayanan R, Kamsa-ad S, Parkin DM and Vatanasapt V (1995). Cancer survival in Khon Kaen province, Thailand. *Int J Cancer*, **61**: 296–300

Taylor R, Smith D, Hoyer A, Coates M and Mc Credie M (1994) *Breast Cancer in New South Wales (1972–91)*. New South Wales Central Cancer Registry: New South Wales

© Cancer Research Campaign 1997